# Highly Ordered Architecture of MicroRNA Cluster

**DOI:** 10.1155/2013/463168

**Published:** 2013-09-30

**Authors:** Bing Shi, Mingxuan Zhu, Shuang Liu, Mandun Zhang

**Affiliations:** ^1^Department of Geriatric Cardiology, Beijing Military General Hospital, Beijing 100700, China; ^2^The 14th Beijing High School, Beijing, 100020, China; ^3^School of Computer Science and Engineering, Hebei University of Technology, Tianjin 300401, China

## Abstract

Although it is known that the placement of genes in a cluster may be critical for proper expression patterns, it remains largely unclear whether the orders of members in an miRNA cluster have biological insights. By investigating the relationship between expression and orders for miRNAs from the oncogenic miR-17-92 cluster, we observed a highly ordered architecture in this cluster. A significant correlation between miRNA expression level and its placement was revealed. More importantly, the placement of these miRNAs is associated with their dysregulation in cancer. Here, we presented the opinion that miRNA clusters are not arranged randomly but show highly ordered architectures, which may have critical roles in physiology and pathology.

## 1. Introduction

Genes are not randomly located in the genome. It is well known that many genes are organized as gene clusters in the genome, and the placement of the gene members in a cluster may have critical roles in biological insights. A typical example is the famous Hox gene family. The relative location of the gene members in the Hox gene cluster corresponds to an expression pattern along the cephalocaudal axis, which is critical for the proper placement of segment structures of animals during early embryonic development [1]. Furthermore, displacement of Hox members would cause abnormal development.

MicroRNAs (miRNAs) are one class of noncoding RNA genes, which mainly negatively regulate the expression of genes at the posttranscriptional level [2]. Like protein-coding genes, a number of miRNAs are grouped into clusters in the genomes [3]. Increasing evidence has shown that members in an miRNA cluster tend to be coexpressed and play as a whole in various physiological and pathological processes [3–5]. A typical case is the human miR-17-92 cluster, which encodes six miRNA genes (from the start to the end: hsa-mir-17, and hsa-mir-18a, hsa-mir-19a, hsa-mir-20a, hsa-mir-19b-1, hsa-mir-92a-1). This miRNA cluster has been confirmed by a number of researchers to play critical roles in various cancers, such as lung cancer [6], chronic lymphocytic leukaemia [7], retinoblastoma [8], breast cancer, gastrointestinal cancer [9], multiple myeloma [10], renal cell carcinoma [11], B-cell lymphomas [12], medulloblastomas [13, 14], acute lymphoblastic leukemia [15], thyroid cancer [16], and mantle cell lymphoma [17]. Because of its strong oncogenic activity, the miR-17-92 cluster was also named as oncomiR-1. Thus, here we asked whether the placement of miRNAs in an miRNA cluster contributes to determining the miRNA expression patterns by investigating the relationship between expression and placement of miRNAs in the miR-17-92 cluster. 

## 2. Materials and Methods

### 2.1. Data of miRNA Expression

We obtained the miRNA expression profile in normal tissues from the study by Liang et al. [18], obtained the miRNA expression profile of normal ovary and ovarian cancer from the study by Bell et al. [19], and obtained the miRNA expression profile of normal blood and blood of cancer patients from the study by Keller et al. [20].

### 2.2. Statistical Analysis

Statistical computations were performed using *R*, a statistical computing language (http://www.r-project.org/), and the correlations were calculated using Spearman's correlation, a nonparametric method.

## 3. Results and Discussion

### 3.1. The Placement of miRNA Cluster Members Shows Correlations with Their Expression Level

**Figure 1:**
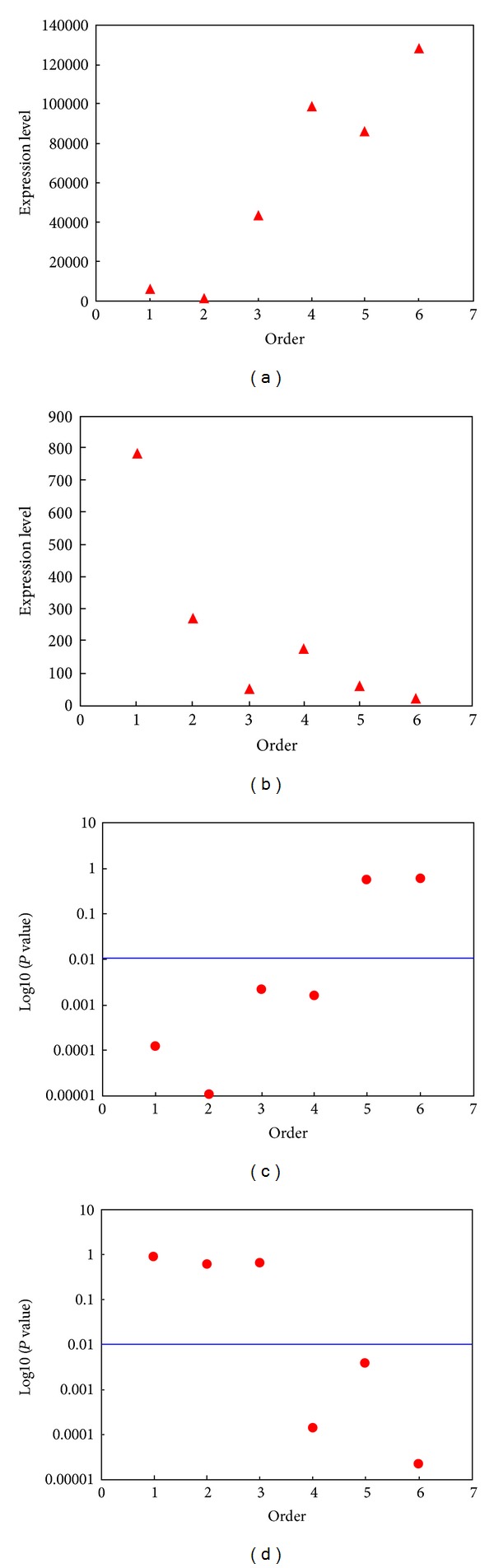
Correlation between the member order of miR-17-92 cluster and their expression. (a) Correlation between the order of mature miRNA and miRNA expression in normal samples; (b) correlation between the order of mature star miRNA and miRNA expression in normal samples; (c) correlation between the order of mature miRNA and the dysregulation significance of these miRNAs in cancer samples; (d) correlation between the order of mature star miRNA and the dysregulation significance of these miRNAs in cancer samples.

We analyzed the relationship between the placement of miRNA-17-92 cluster members and their expression levels using several public datasets. Each of the miRNA genes in this cluster contains two mature miRNAs being denoted as mature and mature star, respectively, that is, miR-17-5p and miR-17-3p (miR-17*). We first calculated the correlation between the miRNA placement and their expression level for the mature miRNAs in normal tissues from Liang et al.'s study [18]. The correlation analysis revealed a significantly positive correlation between the miRNA placement and their expression level (Figure 1(a), *R* = 0.95, *P* = 0.004) for the mature miRNAs. We confirmed this correlation using other two independent datasets, miRNA expression profile in normal ovary (*R* = 0.69, *P* = 0.13) [19] and miRNA expression profile in normal blood (*R* = 0.69, *P* = 0.13) [20]. For the mature star miRNA, because Liang's datasets did not present expression data, we performed correlation analysis based on the other two datasets, as described previously. Interestingly, a reverse pattern was found for the mature star miRNA. The miRNA placement and their expression level show negative correlation (Figure 1(b), *R* = −0.80 and *P* = 0.055) in blood sample dataset and in the ovary sample dataset (*R* = −0.64 and *P* = 0.17).

### 3.2. miRNA Placement in miRNA Clusters Plays Roles in Cancer

We further asked whether the placement of miRNAs in a cluster has roles in pathology. To address this question, we compared the expression level of the six members (both mature miRNAs and mature star miRNAs) of miR-17-92 cluster in ovary cancer samples with that in normal ovary samples. As a result, the significance (*P* value) shows a positive correlation (Figure 1(c), *R* = 0.83, *P* = 0.04) with the placement for the mature miRNAs, whereas the significance shows a negative correlation (Figure 1(d), *R* = −0.92, *P* = 0.01) with the placement for the mature star miRNAs. Furthermore, only the starting four mature miRNAs are significantly upregulated in ovary cancer samples (*t*-test, *P*  values < 0.01); whereas for mature star miRNAs, only the ending three are significantly upregulated in ovary cancer samples (*t*-test, *P*  values < 0.01). These results provide evidence that the placement of miRNA members of an miRNA cluster is correlated with the dysregulation of these miRNAs in cancer, suggesting that the placement of miRNA members of a cluster may have roles in pathological processes.

## 4. Discussion

In summary, we found that the placement of miRNA members of an miRNA cluster has roles in determining the expression patterns of these miRNAs in both physiological and pathological processes. Furthermore, the mature miRNA and miRNA star miRNAs show different patterns. These results suggest that different members should have different biological functions and roles. Indeed, different researchers have obtained different observations. Using the *E*μ*-myc* model of mouse B-cell lymphoma, Olive et al. investigated the tumorigenic potential of the six members of the miR-17-92 cluster. As a result, they found that miR-19 is the key oncogenic components of the cluster [21]. Interestingly, however, in another research, Tsuchida et al. found that miR-92 is the key oncogenic component of the miR-17-92 cluster in colon cancer [22]. Although different members are identified as key oncogenic component of the miR-17-92 cluster by different researchers, the difference could be interpreted using the above observations (Figures 1(c) and 1(d)). In a recent study, Chaulk et al. revealed that the pri-miRNA of miR-17-92 cluster shows a globular tertiary structure. They found that miR-92 is internalized within the core of the folded structure, which leads to a less efficiency in miR-92 biogenesis [23]. This finding is interesting and can interpret the reason why miR-92a* (miR-92a-5p) is in a low expression level in normal tissue and is upregulated in cancer but cannot interpret the reason why miR-92a is in high expression level in normal tissue and does not show upregulation in cancer. Finally, although a correlation between the placement of miRNA members in a cluster and their expression patterns in normal and cancer samples was revealed, the reason and role of this correlation in physiological and pathological processes still remain to be answered.
